# The longitudinal relationship between psychological symptoms and social functioning in displaced refugees

**DOI:** 10.1017/S0033291724003519

**Published:** 2025-02-12

**Authors:** Angela Nickerson, Gulsah Kurt, Belinda Liddell, David Keegan, Randy Nandyatama, Atika Yuanita, Rizka Argadianti Rachmah, Joel Hoffman, Shraddha Kashyap, Natalie Mastrogiovanni, Vivian Mai, Anna Camilleri, Dessy Susanty, Diah Tricesaria, Hasti Rostami, Jenny Im, Marta Gurzeda, Mitra Khakbaz, Sarah Funnell, Zico Pestalozzi, Philippa Specker

**Affiliations:** 1 School of Psychology, University of New South Wales, Sydney, Australia; 2 School of Psychological Sciences, University of Newcastle, Newcastle, Australia; 3 HOST International, Parramatta, NSW, Australia; 4 School of Social Work, Excelsia University College, Macquarie Park, NSW, Australia; 5 Department of International Relations, Gadjah Mada University Yogyakarta, Yogyakarta, Indonesia; 6 SUAKA, Indonesian Civil Society Network for Refugee Rights Protection, Jakarta Pusat, Indonesia; 7 Sydney School of Medicine, Faculty of Medicine and Health, The University of Sydney; 8 School of Social Sciences, Monash University, Melbourne, Australia; 9 Bilya Marlee School of Indigenous Studies, University of Western Australia, Perth, Australia

**Keywords:** refugees, asylum-seekers, displacement, social functioning, mental health, posttraumatic stress disorder

## Abstract

**Background:**

Refugee experiences of trauma and displacement can significantly disrupt established social networks. While social functioning has been routinely associated with mental health, to our knowledge, no study has tested the direction of influence between social and psychological functioning within displaced refugee communities. This study investigated the temporal association between psychological symptoms (PTSD, depression, anger) and multiple facets of social functioning (including community connectedness, perceived social responsibility, positive social support and negative social support).

**Method:**

A culturally diverse sample of refugees (N = 1,235) displaced in Indonesia completed an online survey at four time-points, six months apart. Longitudinal structural equation modelling was used to investigate the temporal ordering between psychological symptoms and social functioning.

**Results:**

Findings revealed that greater psychological symptoms were associated with a subsequent deterioration in social functioning (decreased positive social support and community connectedness and increased negative social support and perceived social responsibility). Greater perceived social responsibility was also associated with subsequent increases in psychological symptoms, while positive social support and community connectedness were bi-directionally associated over-time.

**Conclusions:**

These findings highlight the potential utility of mental health interventions for displaced refugees as a means to improve social functioning and inclusion with host communities. Findings have important implications in guiding the development of interventions and allocation of resources to support refugee engagement and wellbeing in displacement contexts.

Refugees and asylum-seekers are forced to flee their homes due to war, conflict, and persecution. The mental health impact of these experiences is often compounded by significant adversity encountered during and following displacement. Accordingly, approximately one in three refugees and asylum-seekers have probable diagnoses of posttraumatic stress disorder (PTSD) and depression (Henkelmann et al., 2020; Patanè et al., 2022; Steel et al., 2009). In addition to psychological sequelae, forced displacement disrupts social networks, with refugees being compelled to leave behind family and friends when seeking safety in a new country (Liddell, Byrow et al., 2021; Nickerson et al., 2010). Indeed, experiences of loneliness, disconnection, isolation have all been documented amongst refugees (Kurt et al., 2023; Liddell, O’Donnell et al., 2021; Liddell et al., 2022; Nguyen, Slewa-Younan, & Rioseco, 2023; Strang & Quinn, 2021). Notably, isolation has been linked to poorer mental health outcomes in refugees (Chen, Hall, Ling, & Renzaho, 2017). Further, there is substantial cross-sectional evidence that poorer social functioning (i.e., the capacity to form and maintain good-quality relationships and participate in social activities (Lahiri, van Ommeren, & Roberts, 2017) is associated with worse mental health outcomes amongst refugees (Al-Adhami, Berglund, Wångdahl, & Salari, 2022; Berthold et al., 2019; Çankaya, Alan Dikmen, & Dereli Yılmaz, 2020; Haer, Scharpf, & Hecker, 2021; Kurt et al., 2023; Pak, Yurtbakan, & Acarturk, 2023; Schick et al., 2016; Yildirim, Isik, Firat, & Aylaz, 2020). This is problematic as good social functioning is critical to successfully navigating the post-displacement environment (Wachter et al., 2021). This may be especially important for refugees who are displaced in low-resource transit settings (such as low-and-middle income countries), where individuals may rely on social networks in the absence of formal supports to meet basic needs (Posselt et al., 2019).

To date, however, the temporal ordering of psychological symptoms and social functioning in refugees is unclear. Understanding the direction of influence between psychological symptoms and social functioning is important for identifying the most powerful point of intervention to facilitate overall wellbeing, particularly in low-resource settings. One hypothesized direction of influence is that greater social functioning predicts improvements in mental health over time. This has been elaborated in social causation models, such as the stress buffering hypothesis (Cohen & Wills, 1985) and the social support deterioration model (Kaniasty & Norris, 1993). These models propose that social support – the practical or emotional support obtained through interpersonal connections (Wachter et al., 2021) – is an important antecedent to mental health in the aftermath of trauma exposure, such that varying levels of social support may protect against or exacerbate psychological symptoms following a traumatic event. An alternative possibility is that psychological symptoms temporally precede changes in social support. This would be consistent with the social selection and social erosion models, which posit that PTSD symptoms may lead to reduced availability and quality of social support over time due to the individual withdrawing from and/or pushing away important potential sources of support (King et al., 2006). It is also possible that the social selection and social causation models can co-exist. Consistent with this, longitudinal evidence from studies with non-refugee trauma survivors have found support for both the social causation and social erosion models, with a recent meta-analysis finding a bidirectional relationship between social support and PTSD symptoms (Wang et al., 2021).

Psychological models considering the temporal ordering between social functioning and psychological symptoms tend to focus on the relationship between *perceived positive social support* and mental health. There are, however, other important facets of social functioning that are especially relevant to the refugee experience and may influence or be influenced by mental health. For example, *connectedness with the host community* may be important for refugees to foster a sense of belonging in their new home (Ager & Strang, 2008; Kurt et al., 2023). Further, refugees may feel a strong sense of *social responsibility* for family or friends who are living with them in the transit context or in another country and requiring financial, logistical or emotional assistance (Liddell et al., 2020). In addition, perceived social support may not always be positive in nature, with *perceived negative social support* (e.g. the experience of conflict, tensions, and demands from others) being linked to psychological symptoms in the aftermath of exposure to trauma (Andrews, Brewin, & Rose, 2003; Nickerson et al., 2017; Zoellner, Foa, & Brigidi, 1999). In summary, social functioning is multi-faceted; it is thus plausible that the association with psychological symptoms may differ across specific aspects of social functioning.

To date, however, understanding of the longitudinal association between psychological symptoms and social functioning in refugees is limited. One study undertaken with resettled refugees found that social integration stressors predicted the worsening of psychological distress over three years, although the inverse relationship was not tested (Nguyen, Slewa-Younan, & Rioseco, 2023). A second study with resettled refugees found no association between social engagement and mental health outcomes over time, however the measure used to index social engagement (a count of the number of types of individuals/groups participants were engaged with), may have lacked sensitivity to detect these relationships (Nickerson et al., 2022). This relative scarcity of evidence uncovers an important gap in our knowledge – namely understanding the direction of influence between psychological symptoms and social functioning amongst refugees. The current study aimed to address this research gap by investigating the temporal ordering between key indicators of social functioning and psychological symptoms amongst a sample of refugees living in protracted displacement in Indonesia. Indonesia does not permanently resettle refugees, thus all refugees in Indonesia are living in this country temporarily, awaiting resettlement to other countries, although in practicality, this waiting period is often indefinite due to limited resettlement places (Curby, 2020). During this protracted displacement, refugees in Indonesia do not have rights to work or to reunite with family and have limited access to government-sponsored services. This might also increase reliance on social networks to navigate their needs.

Based on theoretical models of social support and mental health following trauma, and research undertaken with trauma-affected populations to date, we hypothesized a bidirectional relationship between social functioning and psychological symptoms over time, consistent with both social selection and social erosion models. Specifically, we predicted that greater positive social support and connectedness with the host community, as well as lower negative social support and perceived social responsibility, would be associated with decreases in psychological symptoms over time. We also predicted that greater psychological symptoms would be associated with subsequent decreases in positive social support and connectedness with the host community, and subsequent increases in negative social support and perceived social responsibility.

## Method

### Participants

Participants were refugees from Arabic, Farsi, Dari, Somali or English-speaking backgrounds living in Indonesia. Refugees from these language groups represented over 80% of refugees living in Indonesia in 2018 at the outset of the study (UNHCR Indonesia, 2018). Participants were recruited via advertisements in refugee services and community-based organizations, referrals, social media and snowball sampling (Sadler, Lee, Lim, & Fullerton, 2010). Participants were required to (1) have a refugee or asylum-seeking background, (2) be literate in written Arabic, Farsi, Dari, Somali or English, (3) be over 18 years of age, and (4) have arrived in Indonesia in January 2013 or later.

Demographic information of participants is presented in Table 1. In this study, 1,235 participants completed the survey at Time-point 1 (T1), 968 at T2, 779 at T3 and 753 at T4, with data being collected six months apart (61.0% retention rate from T1 to T4). Compared to participants who completed T1 only, those who completed all time-points had been in Indonesia longer (T1 only mean = 4.31 years, *SD* = 1.44 vs all time-points mean = 4.80 years, *SD* = 1.81, *t*(171.385) = 5.20, *p* < .001), were more likely to have completed the survey in Dari (T1 only = 16.2% vs all time-points = 24.2%) than English (T1 only = 22.6% vs all time-points = 14.2%, *χ^2^*(4) = 34.53, *p* < .001), reported greater positive social support (T1 only mean = 3.25, *SD* = 0.82 vs all time-points mean = 3.41, *SD* = 0.84, *t*(1114) = 0.31, *p* < .001) and reported greater perceived social responsibility (T1 only mean = 3.01, *SD* = 0.95 vs all time-points mean = 3.22, *SD* = 0.91, *t*(1154) = 0.37, *p* < .001). There were no T1 differences in these groups in gender, age, exposure to potentially traumatic events (PTEs), negative social support, or symptoms of PTSD, depression or anger.

**Table 1.** Participant characteristics

	Mean/N	SD/%
Age (yrs)	30.53	9.07
Gender (Female)	348	28.00%
Language		
Arabic	371	29.80%
English	216	17.50%
Farsi	226	18.20%
Somali	162	13.00%
Dari	260	20.90%
Country of birth		
Afghanistan	440	35.69%
Iraq	259	21.01%
Somalia	187	15.17%
Iran	92	7.46%
Sudan	73	5.92%
Pakistan	49	3.97%
Nigeria	22	1.78%
Ethiopia	17	1.38%
Liberia	15	1.22%
Other	79	6.40%
Education		
Little or no education	162	13.20%
Completed primary school	374	30.48%
Completed high school	424	34.56%
Completed university	178	14.51%
Completed other training	89	7.25%
Marital status		
Married or in a relationship	565	45.97%
Not in a relationship	664	54.03%
Family status		
No immediate family in Indonesia	650	53.50%
Some immediate family in Indonesia	254	20.90%
All immediate family in Indonesia	310	25.50%
Time in Indonesia (yrs)	5.10	1.62
Exposure to potentially traumatic events (PTEs)	7.75	5.02

*Note*: *N* = 1,235.

### Measures

All measures were translated and blind-back translated by accredited translators. Measures were pilot-tested with refugees from each language group with varying levels of education.

***Demographics.*** Demographic information collected included age, gender, language, country of birth, education, marital status and years lived in Indonesia. Exposure to PTEs was indexed using the 16-item Harvard Trauma Questionnaire (Mollica et al., 1992) and 3 additional items relating to natural disaster, physical assault or exposure to serious accident, fire, or explosion (Nickerson et al., 2023). A total count of the number of different PTEs that each participant had experienced and/or witnessed was derived for the present study.

***Psychological symptoms.*** PTSD symptoms were indexed using an adapted 20-item version of the Posttraumatic Diagnostic Scale for DSM-IV (Foa, Riggs, Dancu, & Rothbaum, 1993), with four items added (persistent negative beliefs, distorted blame, persistent negative emotional state, and risk-taking behaviour) and one item removed (sense of a foreshortened future) to reflect the updated DSM-5 criteria. Items were scored on a four-point Likert scale (1 = *Not at all/only one time* to 4 = *5 or more times a week/almost always).* Depression symptoms were measured using the 8-item Patient Health Questionnaire (Kroenke et al., 2009). Items were scored on a four-point Likert scale (1 = *Not at all* to 4 = *nearly every day).* Anger symptoms were measured using the 5-item Dimensions of Anger Reactions scale (Forbes et al., 2014). Items were scored on a five-point Likert scale (1 = *None or almost none of the time* to 5 = *all or almost all of the time).* All scales showed strong internal consistency at all time-points (PTSD: α = 0.95–0.97; Depression: α = 0.89–0.93; Anger: α = 0.89 to 0.92). In this study, means of PTSD, depression and anger symptoms formed the three indicators for the psychological symptoms latent variable at each time-point.

***Positive social support.*** Positive social support was measured using an 8-item scale adapted from social support questions used by Araya, Chotai, Komproe, and de Jong (2007). Items (presented in Table 2) measured the extent to which an individual perceived that they had others they could rely on and talk to. Items were scored on a five-point Likert scale (1 = *Strongly disagree*, 5 = *Strongly agree*). In this study, individual items were used as indicators for the positive social support latent variable at each time-point.

**Table 2.** Factor loadings in metric invariance model for confirmatory factor analysis at time 1

	Unstandardized factor loading	SE	Standardized factor loading	t	p
**Psychological symptoms**					
PTSD symptoms	1.00	0.00	0.87–0.91	–	–
Depression symptoms	1.05	0.02	0.84–0.88	62.69	<.001
Anger reactions	1.12	0.02	0.78–0.80	53.98	<.001
**Positive social support**					
There are people I can depend on to help me if I really need it.	1.00	0.00	0.65–0.71	–	–
I have close relationships that provide me with a sense of emotional security and well-being.	1.09	0.03	0.76–0.80	42.34	<.001
There is someone I could talk to about important decisions in my life.	1.20	0.03	0.79–0.84	43.80	<.001
I have relationships where my competence and skills are recognized.	1.05	0.03	0.73–0.78	40.71	<.001
There is a trustworthy person I could turn to for advice if I were having problems.	1.17	0.03	0.77–0.84	43.07	<.001
I feel strong emotional bond with at least one other person.	1.00	0.03	0.65–0.71	36.96	<.001
There are people who admire my talents and abilities.	0.98	0.03	0.69–0.74	38.10	<.001
There are people I can count on in an emergency.	1.11	0.03	0.75–0.80	42.16	<.001
**Negative social support**					
How often did family or friends criticize you?	1.00	0.00	0.77–0.86	–	–
How often did family or friends create tensions or arguments with you?	0.95	0.03	0.75–0.81	36.35	<.001
**Perceived social responsibility**					
I feel responsible for other people.	1.00	0.00	0.76–0.78	–	–
Other people rely on me.	1.03	0.03	0.82–0.86	40.30	<.001
If I wasn’t here, people would suffer.	0.66	0.02	0.52–0.55	28.22	<.001

***Negative social support.*** Negative social support was measured using three items that were adapted from the Schuster Social Support Questions (Schuster, Kessler, & Aseltine, 1990). These items indexed how often the participants had negative interactions with family and friends. One item (“*how often did family or friends make too many demands on you?*”) was removed from the scale due to a low factor loading and conceptual overlap with the Perceived Social Responsibility Scale (see Results). The remaining two items are presented in Table 2. Items were scored on a four-point Likert scale (1 = *Never*, 4 = *Extremely often*). In this study, individual items were used as indicators for the negative social support latent variable at each time-point.

***Perceived social responsibility.*** Perceived social responsibility was indexed using three items developed for this study (see Table 2). These items were scored on a five-point Likert scale (1 = *Strongly disagree*, 5 = *Strongly agree*). In this study, individual items were used as indicators for the perceived social responsibility latent variable at each time-point.

***Connectedness with the Indonesian community.*** Connectedness with the Indonesian community was indexed using a single item developed for this study (see Table 2). This was measured on a 5-point Likert scale (1 = *Not at all connecte*d, 5 = *Extremely connected*).

### Procedure

T1 data was collected between February and October 2020. Data for T2 to T4 was collected at six-month intervals following completion of T1. Participants initially registered their interest in participating on the study website where they completed eligibility screening questions. Eligible participants completed online informed consent procedures and were sent a personalized study link. Measures were administered via the KeySurvey platform in the participant’s preferred language (English, Arabic, Dari, Somali, Farsi). Participants received a IDR100,000 ($USD7) shopping voucher for taking part in each time-point of the study. This study was approved by UNSW Human Research Ethics Committee (HC190494) and Atma Jaya University, Jakarta (0792/III/LPPM-PM.10.05/07/2019).

### Data analysis

We used longitudinal structural equation modelling in MPlus version 8 (Muthen & Muthen, 1998) to investigate the association between psychological symptoms and different facets of social functioning over time. Full information maximum likelihood estimation was used to account for missing data on endogenous variables by adjusting model parameters based on the available information. Model fit was evaluated using the following indices: comparative fit index (CFI) > 0.95, Tucker-Lewis Index (TLI) > 0.95, standardized root mean square residual (SRMR) < 0.08, and root mean square error of approximation (RMSEA) < 0.06 (Hu & Bentler, 1999). Where relevant, we compared nested models using a chi-square difference test.

First, we used confirmatory factor analysis (CFA) to evaluate the measurement model. For this model, we investigated the fit of latent variables and their various indicator variables at each time-point in a single model. The latent variable of psychological symptoms was indexed by three indicator variables, namely mean PTSD, depression and anger symptom scores; positive social support was indexed by eight indicator items; negative social support was indexed by two indicator items, and perceived social responsibility was indexed by three indicator items. Connection to the Indonesian community was indexed by a single item, and thus was represented by an observed variable and not included in the measurement model. We elected to include multiple latent variables for social functioning (compared to a single latent variable for mental health) as we were most interested in investigating the association between overall mental health and specific types of social functioning over time. After estimating an unconstrained CFA model that included all latent and indicator variables at all time-points, we evaluated measurement invariance across time by estimating increasingly constrained models (Little, 2013). Model fit was progressively tested and compared to obtain the most parsimonious measurement model. First, we estimated a baseline or unconstrained model. Next, we tested metric invariance, where factor loadings were constrained to equality at each time-point, followed by scalar invariance where intercepts were also constrained to equality over time-points. Next, we tested for residual invariance where residual errors were set to equality over time-points, and finally we tested factor invariance where the variance of factors were set to 1. Each model was compared to the previous model, and model fit determined by the following indices: CFI > 0.95, TLI > 0.95, SRMR<0.08, RMSEA<0.06 (Hu & Bentler, 1999).

Next, we tested the structural model, retaining the measurement model structure from the previous step that balanced parsimony and model fit. The structural model investigated the association between psychological symptoms, positive social support, negative social support, perceived social responsibility and connection with the Indonesian community across time-points. Pathways that were modelled between each time-point included autoregressive and cross-lagged paths. When evaluating the structural model, we again sought to optimise parsimony. Accordingly, we first tested an unconstrained model where all structural pathways (e.g., pathways between latent variables) were allowed to vary freely over time. Next, we tested a model where autoregressive pathways between variables were constrained to equality between time-points. We then tested a model where cross-lagged pathways between variables were constrained to equality between time-points. Finally, we tested a model where residual covariances between latent variables were constrained to equality between time-points.

After establishing the optimal model, we included covariates in the model to control for the effects of variables known to predict mental health and social functioning outcomes, including age, gender, language group (which was considered a proxy for cultural group), time in Indonesia, and exposure to potentially traumatic events. All T1 variables were regressed on covariates. We used multiple imputation (20 datasets) to account for missing data on covariates.

## Results

### Measurement model

Testing of the baseline (configural invariance) model revealed adequate model fit: CFI = 0.88, TLI = 0.87, RMSEA = 0.04, SRMR = 0.05. Inspection of factor loadings revealed that one of the items indexing negative social support (*“How often did family or friends make too many demands on you?”*) yielded standardized factor loadings of <0.50 at two time-points (T1 = 0.44, T2 = 0.48, T3 = 0.52, T4 = 0.57), which indicates that this item did not load adequately onto the negative social support factor (Taherdoost, Sahibuddin, & Jalaliyoon, 2022). Further, this item overlapped with the Perceived Responsibility Scale. We thus removed this item and re-tested the baseline configural invariance model. This model showed slightly improved model fit: CFI = 0.89, TLI = 0.88, RMSEA = 0.04, SRMR = 0.04. Subsequent models omitted this item. Next, we tested metric invariance where factor loadings were constrained to equality over time within each latent variable. Model fit was not significantly different to the configural invariance model: χ^2^diff (36) = 38.51, *p* = .657, CFI = 0.89, TLI = 0.88, RMSEA = 0.04, SRMR = 0.04. Thus, we retained the metric invariance model. We then tested scalar variance, where intercepts were constrained to equality over time within each latent variable. This model showed significantly poorer fit to the metric invariance model: χ^2^diff (48) = 81.71, *p* = .002, CFI = 0.89, TLI = 0.88, RMSEA = 0.04, SRMR = 0.04. Accordingly, the metric invariance model was retained for subsequent analyses. Unstandardized and standardized factor loadings for this model are presented in Table 2.

### Structural model

The unconstrained structural model revealed adequate fit: CFI = 0.875, TLI = 0.871, RMSEA = 0.040, SRMR = 0.072. Constraint of autoregressive paths to equality between time-points revealed a small but significant change in model fit according to the chi-square difference statistic: χ^2^diff (10) = 18.73, *p* = .044, however other indices revealed slight improvements in fit: CFI = 0.879, TLI = 0.871, RMSEA = 0.040, SRMR = 0.066. Similarly, constraint of cross-lagged paths to equality between time-points revealed a small but significant change in model fit compared to the autoregressive constrained model according to the chi-square difference statistic: χ^2^diff (40) = 56.16, *p* = .046. Again, other indices revealed little to no change in model fit: CFI = 0.878, TLI = 0.873, RMSEA = 0.039, SRMR = 0.067. Constraint of residual error variances to equality between time-points revealed a significant change in model fit according to the chi-square difference statistic: χ^2^diff (26) = 226.62, *p* < .001, and small decreases in model fit according to other indices: CFI = 0.875, TLI = 0.871, RMSEA = 0.040, SRMR = 0.072. Theorists have noted that the chi-square can be sensitive to trivial influences in the case of large sample sizes (Little, 2013), and thus yield assessments of model fit that are too conservative. Given that (1) other model indices revealed no change in fit, (2) the change in chi-square was close to the 0.05 *p*-value in the instance of the constrained autoregressive and cross-lagged paths, and (3) the goal was to optimise model parsimony, we elected to retain the model with autoregressive and cross-lagged pathways constrained between time-points. We considered retaining the model with residual error variances constrained to equality between time-points. However, while this model yielded a highly significant chi-square difference statistic, (small) decreases in model fit in other indices were observed. We thus elected to allow error variances to vary between time-points and retained the model that only constrained autoregressive and cross-lagged paths.

Model parameters are presented in Table 3, and residual covariances between latent variables are presented in Supplementary Table 1.

**Table 3.** Model parameters in structural model

Predictor	Outcome	B	SE	Beta	t	p
Autoregressive paths						
T1 PSS➔	T2 PSS	0.51	0.02	0.49 to 0.50	21.90	<.001
T1 NSS ➔	T2 NSS	0.58	0.03	0.52 to 0.61	19.85	<.001
T1 PSR ➔	T2 PSR	0.61	0.03	0.66 to 0.66	23.52	<.001
T1 Connection with Indonesian Community ➔	T2 Connection with Indonesian Community	0.57	0.02	0.56 to 0.59	33.80	<.001
T1 Psychological symptoms ➔	T2 Psychological Symptoms	0.86	0.02	0.80 to 0.83	43.53	<.001
Cross-lagged paths						
T1 PSS ➔	T2 NSS	−0.02	0.02	−0.02 to −0.03	−1.13	0.257
T1 PSS ➔	T2 PSR	0.01	0.03	0.01	0.41	0.679
T1 PSS ➔	T2 Connection with Indonesian Community	0.10	0.03	0.08	3.88	<.001
T1 PSS ➔	T2 Psychological symptoms	0.01	0.02	0.01	0.59	0.559
T1 NSS ➔	T2 PSS	−0.01	0.03	−0.01	−0.30	0.765
T1 NSS ➔	T2 PSR	0.01	0.04	0.01	0.17	0.868
T1 NSS ➔	T2 Connection with Indonesian Community	0.05	0.03	0.03 to 0.04	1.45	0.146
T1 NSS ➔	T2 Psychological symptoms	−0.01	0.02	−0.01	−0.09	0.993
T1 PSR ➔	T2 PSS	0.03	0.02	0.03	1.40	0.162
T1 PSR ➔	T2 NSS	0.03	0.02	0.039	1.51	0.130
T1 PSR ➔	T2 Connection with Indonesian Community	0.01	0.02	0.01	0.51	0.608
T1 PSR ➔	T2 Psychological symptoms	0.04	0.01	0.05	2.48	0.013
T1 Connection with Indonesian Community ➔	T2 PSS	0.06	0.02	0.07	3.79	<.001
T1 Connection with Indonesian Commnity ➔	T2 NSS	0.02	0.01	0.03 to 0.04	1.75	0.081
T1 Connection with Indonesian Commnity ➔	T2 PSR	0.02	0.02	0.02 to 0.03	1.34	0.181
T1 Connection with Indonesian Commnity ➔	T2 Psychological Symptoms	0.02	0.01	0.02	1.54	0.124
T1 Psychological symptoms ➔	T2 PSS	−0.06	0.03	−0.05	−2.03	0.042
T1 Psychological symptoms ➔	T2 NSS	0.18	0.03	0.17 to 0.18	7.11	<.001
T1 Psychological symptoms ➔	T2 PSR	0.10	0.03	0.07 to 0.08	3.06	0.002
T1 Psychological symptoms ➔	T2 Connection with Indonesian Community	−0.08	0.03	−0.06	−2.72	0.006
Covariates						
Gender ➔	T1 PSS	0.02	0.05	0.012	0.41	0.682
Gender ➔	T1 NSS	0.00	0.05	0.003	0.09	0.926
Gender ➔	T1 PSR	0.05	0.06	0.024	0.79	0.428
Gender ➔	T1 Connection with Indonesian Community	−0.33	0.06	−0.153	−5.34	<.001
Gender ➔	T1 Psychological symptoms	0.08	0.04	0.056	2.00	0.046
Age ➔	T1 PSS	0.01	0.00	0.091	2.97	0.003
Age ➔	T1 NSS	0.00	0.00	−0.047	−1.39	0.163
Age ➔	T1 PSR	0.01	0.00	0.1	3.27	0.001
Age ➔	T1 Connection with Indonesian Community	−0.01	0.00	−0.102	−3.56	<.001
Age ➔	T1 Psychological symptoms	0.00	0.00	−0.006	−0.20	0.84
English ➔	T1 PSS	−0.34	0.07	−0.174	−5.05	<.001
English ➔	T1 NSS	0.15	0.06	0.091	2.41	0.016
English ➔	T1 PSR	−0.09	0.08	−0.038	−1.10	0.27
English ➔	T1 Connection with Indonesian Community	0.21	0.08	0.08	2.48	0.013
English ➔	T1 Psychological symptoms	0.44	0.05	0.257	8.09	<.001
Farsi ➔	T1 PSS	0.05	0.07	0.026	0.77	0.443
Farsi ➔	T1 NSS	0.24	0.06	0.154	4.05	<.001
Farsi ➔	T1 PSR	−0.34	0.09	−0.122	−3.58	<.001
Farsi ➔	T1 Connection with Indonesian Community	0.49	0.08	0.191	5.93	<.001
Farsi ➔	T1 Psychological symptoms	0.39	0.05	0.23	7.21	<.001
						<.001
Somali ➔	T1 PSS	−0.56	0.08	−0.249	−7.24	<.001
Somali ➔	T1 NSS	−0.19	0.07	−0.104	−2.74	0.006
Somali ➔	T1 PSR	0.61	0.08	0.255	7.49	<.001
Somali ➔	T1 Connection with Indonesian Community	−0.12	0.10	−0.041	−1.26	0.208
Somali ➔	T1 Psychological symptoms	−0.02	0.06	−0.009	−0.26	0.793
Dari ➔	T1 PSS	−0.09	0.07	−0.046	−1.32	0.188
Dari ➔	T1 NSS	0.18	0.06	0.117	2.98	0.003
Dari ➔	T1 PSR	0.70	0.08	0.31	8.80	<.001
Dari ➔	T1 Connection with Indonesian Community	0.16	0.08	0.066	1.99	0.047
Dari ➔	T1 Psychological symptoms	0.33	0.05	0.21	6.42	<.001
Time in Indonesia ➔	T1 PSS	0.00	0.01	−0.008	−0.25	0.805
Time in Indonesia ➔	T1 NSS	−0.04	0.01	−0.091	−2.65	0.008
Time in Indonesia ➔	T1 PSR	0.03	0.02	0.056	1.82	0.069
Time in Indonesia ➔	T1 Connection with Indonesian Community	−0.03	0.02	−0.049	−1.68	0.093
Time in Indonesia ➔	T1 Psychological symptoms	−0.01	0.01	−0.032	−1.10	0.273
PTE exposure ➔	T1 PSS	0.00	0.01	0.005	0.16	0.876
PTE exposure ➔	T1 NSS	0.03	0.00	0.204	5.83	<.001
PTE exposure ➔	T1 PSR	0.02	0.01	0.094	2.85	0.004
PTE exposure ➔	T1 Connection with Indonesian Community	0.00	0.01	0	−0.01	0.994
PTE exposure ➔	T1 Psych symptoms	0.05	0.00	0.41	14.06	<.001

*Note*: Gender was coded where 0, male and 1, female.

Abbreviations: PSS , Positive Social Support; NSS, Negative Social Support; PSR, Perceived Social Responsibility; PTE, Potentially Traumatic Events.

***Associations between psychological symptoms and subsequent social functioning.*** Findings revealed that psychological symptoms were associated with subsequent changes in all social functioning variables over time including decreases in positive social support and connection with the Indonesian community and increases in negative social support and perceived social responsibility.

***Associations between social functioning and subsequent psychological symptoms.*** Findings revealed that greater perceived social responsibility was associated with subsequent increases in psychological symptoms. No other facet of social functioning (i.e., community connectedness, positive social support and negative social support) was associated with subsequent changes in psychological symptoms.

***Associations between different types of social functioning over time.*** Greater connection with the Indonesian community was associated with subsequent increases in positive social support and vice versa.

## Discussion

This study was the first, to our knowledge, to investigate the temporal association between psychological symptoms and multiple facets of social functioning in refugees. A key finding of this study (consistent with our hypotheses) was that greater psychological symptoms were associated with subsequent changes in social functioning, including decreased positive social support and connectedness with the host community and increased negative social support and perceived social responsibility. Contrary to our hypotheses, this temporal relationship was unidirectional with the exception of perceived social responsibility, which was bi-directionally associated with psychological symptoms.

These findings highlight the pivotal role of mental health in contributing to social functioning amongst refugees living in a transit setting. The results are consistent with social selection and social erosion models that posit an association between mental health and social support in the aftermath of traumatic events (King et al., 2006). These models suggest that social resources, such as the availability and quality of social support, reduces over time as psychological symptoms cause an individual to withdraw from and/or push away others (King et al., 2006; Wang et al., 2021). Our findings build on these models to suggest that psychological symptoms are associated with not only subsequent reductions in positive social supports and community connectedness, but also with increased negative social support.

In contrast, these findings are not in line with social causation models of mental health, which argue that reduced social support leads to exacerbated psychological symptoms over time (Cohen & Wills, 1985; Kaniasty & Norris, 1993). One possible explanation for this finding is that differential associations between mental health and social functioning may be observed across contexts. This is consistent with contemporary models of wellbeing which focus on the interaction between the individual’s environmental circumstances, psychological skills and social context (Bonanno, Chen, & Galatzer-Levy, 2023; Craske, Herzallah, Nusslock, & Patel, 2023; Kashyap et al., 2021). Specifically, a unidirectional association between mental health and social functioning (with the exception of perceived social responsibility) may be more likely in contexts of impermanence, such as refugee transit settings like Indonesia. In transit contexts, a refugee’s ability to leverage social resources to buffer against mental ill-health is often undermined by notable external factors. For example, in Indonesia, many refugees have travelled alone, or without critical social attachment figures, and thus rebuilding important social connections can be challenging (Liddell et al., 2020; Liddell, Byrow et al., 2021; Liddell et al., 2022). Additionally, government policies that disallow work rights and mainstream schooling serves to discourage integration. At the same time, refugees in transit settings may be reluctant to develop deep social relationships as they do not intend to stay in the transit country permanently (Mixed Migration Center, 2021). This social context differs markedly to that of non-refugee trauma survivors (who represent the samples in most research studies to date) where there may be greater opportunity to establish, and leverage, social networks to support one’s psychological recovery (Wang et al., 2021). Further research investigating differences between the number and types of social connections, and their associations with mental health across displacement contexts (e.g. transit vs permanent resettlement settings) would shed light on this issue.

It is notable that greater perceived social responsibility was bidirectionally associated with increased psychological symptoms over time in this study. Perceived social responsibility in this study was operationalized as feeling responsible for other people, including concern that others would suffer in the absence of the participant. This finding highlights the substantial social burden experienced by many refugees living in transit settings (Liddell et al., 2020). Anecdotal reports from participants in this study revealed that many participants were financially responsible for family members who remained in their country of origin. This is extremely challenging in a context like Indonesia where refugees are denied work rights and access to social security and where resettlement options are very limited. It may be the case that being responsible for others, in a context where an individual’s capacity to fulfill these social responsibilities is very limited, may lead to increased distress. Further, experiencing greater psychological symptoms may impair individuals’ perceived capacity to meet these responsibilities, thus increasing their sense of responsibility and associated distress. This finding points to the importance of structural changes, for example, facilitating permanent resettlement for refugees living in transit settings or, if not possible, increasing the financial and social security of refugees by providing access to essential services, the right to work, and community-building initiatives both within refugee communities and between refugee and host communities. These changes have important implications for alleviating the negative mental health consequences of social burdens often held by refugees in transit settings.

The findings of this study should be interpreted in the context of several limitations. It is notable that our measures of social support in this study focused on *perceived social support* and were thus not objective indicators of social engagement. Perceived social support may be subject to reporting biases, which may have strengthened the association between this variable and psychological symptoms. Future research could index social networks to gain a more nuanced understanding of the type, quantity, and quality of social interactions and their associations with psychological symptoms. Similarly, a more in-depth scale indexing one’s connection to the host community would be preferable instead of a single item. Finally, the sample in this study was a convenience sample. While our sample demonstrated characteristics that were consistent with the United Nations High Commissioner records of refugees in Indonesia (UNHCR Indonesia, 2018), it is unclear how generalizable these results are to other refugees in Indonesia or those displaced to other transit settings.

Findings from this study have important implications for government and non-government organizations supporting refugees living in transit countries. The finding that greater psychological symptoms were associated with subsequent worsening across multiple facets of social functioning highlights the critical importance of providing mental health-focused programs for refugees living in these settings. In low-resource contexts where formal supports are not available, the ability to successfully navigate the social environment is likely to be critical to survival. These findings suggest that supporting psychological recovery among refugees may also confer social benefits. This is consistent with findings from research suggesting that improving psychological symptoms via the implementation of psychological interventions results in improved social functioning and reduced interpersonal-related stressors (De Silva et al., 2013; Renner, Cuijpers, & Huibers, 2014; Spaaij et al., 2023). In many transit settings, there exist very limited mental health services for refugees due to lack of resources to support these programs. These findings suggest that the establishment of these services represents a critical step to support refugees to adapt to their new environments, and to foster social cohesion within societies hosting refugees.

Further, these findings point to the challenges associated with the establishment of social networks for refugees living in transit countries. In low-resource contexts where there may be policies and circumstances that actively discourage the integration of refugees and local communities, the task of developing a social network is very challenging. This is further highlighted by our findings that psychological symptoms make this process even more difficult. It may be the case that difficulty forming social networks reduces the potential down-stream benefits of social engagement on psychological symptoms. If this is the case, greater efforts need to be made to facilitate the development of social networks for refugees living in transit countries.

In conclusion, this study represented the first longitudinal investigation of social functioning and psychological symptoms amongst refugees living in a transit setting. A key finding of this study was that psychological symptoms led to significantly reduced social functioning over time. Findings highlight the importance of the provision of mental health services for refugees living in a state of uncertainty to facilitate positive social outcomes.

## Supporting information

Nickerson et al. supplementary material Nickerson et al. supplementary material

## Data Availability

The datasets used and/or analysed during the current study are available from the corresponding author on reasonable request.

## Supplementary material

The supplementary material for this article can be found at http://doi.org/10.1017/S0033291724003519.
