# Supplementary material for: The longitudinal relationship between psychological symptoms and social functioning in displaced refugees
Source: Psychol Med. 2025 Feb 12;55:e40. doi: 10.1017/S0033291724003519 (PMC12017371; doi:10.1017/S0033291724003519)
Supplement: Nickerson et al. supplementary material [file S0033291724003519sup001.docx]

Supplementary Table A. Residual Covariances between Latent Variables in Structural Model.

|  |  | 1 | 2 | 3 | 4 | 5 | 6 | 7 | 8 | 9 | 10 | 11 | 12 | 13 | 14 | 15 | 16 |
| --- | --- | --- | --- | --- | --- | --- | --- | --- | --- | --- | --- | --- | --- | --- | --- | --- | --- |
| 1 | T1 Psych symptoms | 1.00 |  |  |  |  |  |  |  |  |  |  |  |  |  |  |  |
| 2 | T2 Psych symptoms | 0.75*** | 1.00 |  |  |  |  |  |  |  |  |  |  |  |  |  |  |
| 3 | T3 Psych symptoms | 0.69** | 0.81*** | 1.00 |  |  |  |  |  |  |  |  |  |  |  |  |  |
| 4 | T4 Psych symptoms | 0.65*** | 0.73*** | 0.82*** | 1.00 |  |  |  |  |  |  |  |  |  |  |  |  |
| 5 | T1 PSS | -0.14*** | -0.03 | -0.05 | -0.06 | 1.00 |  |  |  |  |  |  |  |  |  |  |  |
| 6 | T2 PSS | -0.13*** | -0.08* | -0.04 | -0.08 | 0.49*** | 1.00 |  |  |  |  |  |  |  |  |  |  |
| 7 | T3 PSS | -0.13** | -0.13** | -0.31** | -0.10* | 0.47*** | 0.53*** | 1.00 |  |  |  |  |  |  |  |  |  |
| 8 | T4 PSS | -0.08* | -0.08 | -0.04 | -0.08 | 0.46*** | 0.46*** | 0.50*** | 1.00 |  |  |  |  |  |  |  |  |
| 9 | T1 NSS | 0.59*** | 0.41*** | 0.40*** | 0.37*** | -0.07* | -0.08* | -0.03 | -0.05 | 1.00 |  |  |  |  |  |  |  |
| 10 | T2 NSS | 0.45*** | 0.54*** | 0.44*** | 0.35*** | -0.03 | -0.02 | -0.10* | -0.09* | 0.56*** | 1.00 |  |  |  |  |  |  |
| 11 | T3 NSS | 0.44*** | 0.42*** | 0.58*** | 0.43*** | -0.01 | -0.05 | -0.14** | -0.04 | 0.53*** | 0.56*** | 1.00 |  |  |  |  |  |
| 12 | T4 NSS | 0.48*** | 0.43*** | 0.51*** | 0.55*** | -0.09 | -0.07 | -0.13*** | -0.02 | 0.61*** | 0.59*** | 0.72*** | 1.00 |  |  |  |  |
| 13 | T1 PSR | 0.15*** | 0.18*** | 0.18*** | 0.19*** | 0.39*** | 0.22*** | 0.19*** | 0.25*** | 0.14*** | 0.15*** | 0.14** | 0.18*** | 1.00 |  |  |  |
| 14 | T2 PSR | 0.16*** | 0.21*** | 0.19*** | 0.19*** | 0.17*** | 0.38*** | 0.18*** | 0.19*** | 0.11* | 0.21*** | 0.08 | 0.16** | 0.51*** | 1.00 |  |  |
| 15 | T3 PSR | 0.18*** | 0.23*** | 0.24*** | 0.22*** | 0.22*** | 0.29*** | 0.39*** | 0.20*** | 0.17*** | 0.17*** | 0.16** | 0.18*** | 0.59*** | 0.62*** | 1.00 |  |
| 16 | T4 PSR | 0.14*** | 0.19*** | 0.18*** | 0.27*** | 0.27*** | 0.17*** | 0.20*** | 0.45*** | 0.17*** | 0.12* | 0.11* | 0.27*** | 0.53*** | 0.58*** | 0.55*** | 1.00 |

Note: PSS = Positive Social Support; NSS = Negative Social Support; PSR = Perceived Social Responsibility; Psych Symptoms = Psychological Symptoms.
